# Sulforaphane improves the bronchoprotective response in asthmatics through Nrf2-mediated gene pathways

**DOI:** 10.1186/s12931-015-0253-z

**Published:** 2015-09-15

**Authors:** Robert H. Brown, Curt Reynolds, Allison Brooker, Paul Talalay, Jed W. Fahey

**Affiliations:** Department of Anesthesiology and Critical Care Medicine, Johns Hopkins University School of Medicine, Baltimore, MD USA; Division of Pulmonary Medicine and Critical Care, Department of Medicine, Johns Hopkins University School of Medicine, Baltimore, MD USA; Department of Radiology, Johns Hopkins University School of Medicine, Baltimore, MD USA; Lewis B. and Dorothy Cullman Chemoprotection Center, Department of Pharmacology and Molecular Sciences, Johns Hopkins University School of Medicine, Baltimore, MD USA; Department of Environmental Health Sciences, Johns Hopkins University School of Public Health, Room E7614, 615 N. Wolfe Street, Baltimore, MD 21205 USA; Center for Human Nutrition, Department of International Health, Johns Hopkins University School of Public Health, Baltimore, MD USA

**Keywords:** Asthma, Bronchodilation, Oxidative stress

## Abstract

**Background:**

It is widely recognized that deep inspiration (DI), either before methacholine (MCh) challenge (Bronchoprotection, BP) or after MCh challenge (Bronchodilation, BD) protects against this challenge in healthy individuals, but not in asthmatics. Sulforaphane, a dietary antioxidant and antiinflammatory phytochemical derived from broccoli, may affect the pulmonary bronchoconstrictor responses to MCh and the responses to DI in asthmatic patients.

**Methods:**

Forty-five moderate asthmatics were administered sulforaphane (100 μmol daily for 14 days), BP, BD, lung volumes by body-plethsmography, and airway morphology by computed tomography (CT) were measured pre- and post sulforaphane consumption.

**Results:**

Sulforaphane ameliorated the bronchoconstrictor effects of MCh on FEV_1_ significantly (on average by 21 %; p = 0.01) in 60 % of these asthmatics. Interestingly, in 20 % of the asthmatics, sulforaphane aggravated the bronchoconstrictor effects of MCh and in a similar number was without effect, documenting the great heterogeneity of the responsiveness of these individuals to sulforaphane. Moreover, in individuals in whom the FEV_1_ response to MCh challenge decreased after sulforaphane administration, i.e., sulforaphane was protective, the activities of Nrf2-regulated antioxidant and anti-inflammatory genes decreased. In contrast, individuals in whom sulforaphane treatment enhanced the FEV_1_ response to MCh, had increased expression of the activities of these genes. High resolution CT scans disclosed that in asthmatics sulforaphane treatment resulted in a significant reduction in specific airway resistance and also increased small airway luminal area and airway trapping modestly but significantly.

**Conclusion:**

These findings suggest the potential value of blocking the bronchoconstrictor hyperresponsiveness in some types of asthmatics by phytochemicals such as sulforaphane.

## Background

Although corticosteroids are currently the mainstay of asthma treatment, some asthmatics do not respond to corticosteroids even at high doses [1] at which adverse effects impose therapeutic limitations. Moreover, corticosteroids are almost universally effective in controlling many types of asthma, and this obscures the heterogeneity of the disease [2]. Although inflammation may be an important inciting factor, other molecular mechanisms of airway disease may play a crucial role in asthma.

Antioxidant deficiencies have been associated with poor asthma control and accelerated decline of lung function [3–5]. Studies in mice suggest that disruption of the nuclear factor erythroid-related factor 2 (Nrf2) signaling pathway is a possible cause of chronic inflammation, such as that associated with asthma [6]. Nrf2 is responsible for maintaining and restoring cellular homeostasis through antioxidant, anti-inflammatory, and other mechanisms. Thus Nrf2 regulates critical enzymes concerned with the biosynthesis of glutathione (GSH), the dominant, small molecule, intracellular antioxidant. A protective effect of GSH on inflammatory pathologies of the lung including asthma, has been demonstrated in mice [7]. Another potentially critical role of the Nrf2 pathway in asthma is highlighted by the dramatic increase in eosinophilic airway inflammation, airway hyperreactivity, and T helper cell 2 (Th2) cytokine production in Nrf2 knockout mice [6].

Sulforaphane, a phytochemical derived from cruciferous vegetables, is a potent inducer of a variety of cytoprotective (antioxidant and anti-inflammatory) enzymes (reviewed by [8, 9]). It also inhibits the production of nitric oxide, counteracts mitochondrial dysfunction and corrects low oxidative phosphorylation, reduces lipid peroxidation, and upregulates the heat shock response. In many tissues, sulforaphane transcriptionally upregulates the genes encoding cytoprotective enzymes primarily through the Keap1-Nrf2-ARE system [9, 10]. Sulforaphane also potently inhibits inflammation by inhibiting the nuclear factor kappa-light-chain-enhancer of activated B cells (NfκB) cascade [11], the macrophage migration inhibitory factor (MIF), the p38 mitogen-activated protein kinase (MAPK) activation cascade, and by modulating β-catenin signaling [12–14].

Unlike direct antioxidants such as vitamin C, sulforaphane is an indirect antioxidant and upregulates multiple antioxidant pathways without being consumed in the antioxidation process [15]. Sulforaphane boosts the antioxidant capacity of cells by at least two major indirect mechanisms: by induction of phase 2 cytoprotective enzymes and by dramatically increasing cellular GSH levels. Antioxidant deficiencies have been strongly associated with poor asthma control and accelerated lung function decline, and represent a reasonable therapeutic target [16–18].

Nrf2-mediated signaling pathways limit airway eosinophilia, mucus hypersecretion, and airway hyperresponsiveness to allergen challenge in a murine model of asthma [6]. Genetic disruption of the Nrf2 gene (knock-out models) leads to severe allergen-driven airway inflammation and hyper-responsiveness in mice [6]. An et al. [19] recently showed that airway smooth muscle cells isolated from Nrf2^−/−^ mice exhibited significantly higher contractile force compared to cells isolated from Nrf2^+/+^ mice. Thus, there is strong evidence for the role of oxidative stress, mediated through dysfunction of the Nrf2 pathway, as a mechanism of airway hyper-responsiveness characteristic of asthma.

Sulforaphane can be safely and consistently administered to humans by feeding a broccoli sprout extract in which its precursor glucosinolate (glucoraphanin) has been enzymatically hydrolyzed [9]. Sulforaphane has very low toxicity. Its administration is well tolerated and it is extensively consumed as a component of cruciferous vegetables. It may be considered a food, a dietary supplement, or a drug, depending on dosage and its intended use [9, 20, 21].

It has been known for more than 20 years that deep inspiration (DI) protects against MCh induced bronchial constriction. This protective response is characteristically absent in even mild asthmatic patients [22, 23], and indeed, DI can even exacerbate the bronchoconstrictor effects of MCh in asthmatics [24]. The beneficial effects of DIs occurring before or after MCh challenge have been designated bronchoprotection (BP) and bronchodilation (BD), respectively [22, 25]. Elucidation of the pathways leading to the loss of the BP and BD responses in asthma may give further insight into the disease process and reveal new treatments.

The current study was designed with the primary objective to test the hypothesis that sulforaphane could augment the deep inspiration (DI)-induced bronchoprotection (BP) and bronchodilation (BD) responses in individuals with airways hyperresponsiveness. Secondary objectives included testing the hypotheses that sulforaphane would also affect pulmonary function and lung morphology as shown by high resolution CT scans.

## Methods

### Screening

The protocol was approved by the Johns Hopkins IRB and written informed consent was obtained (NA_00011275) and registered at ClinicalTrials.gov (NCT00994604). A total of 51 individuals with airway hyperresponsiveness were screened who reported upper or lower respiratory symptoms (or both) in the absence of upper respiratory infections in the previous 12 months. Five subjects withdrew, and one subject failed the screen. Subjects were also required to have FEV_1_ ≥ 70 % of predicted values, and a positive conventional multi-dose methacholine (MCh) inhalation challenge (PC_20,_ provocative concentration causing a 20 % drop in FEV_1_) of ≤ 25 mg/ml, as well as a BP effect of DI of less than 40 %. Short-acting or long-acting bronchodilators were withheld before MCh challenge for 12 or 48 hours, respectively. The demographic and baseline pulmonary functions of smokers (n = 15) and non-smokers (n = 30) were similar (Table 1). Thirty-one subjects were taking albuterol, 13 were taking fluticasone/salmeterol, 8 were taking fluticasone alone, 3 were taking montelukast, 1 was taking beclomethasone, 1 was taking budesonide/formoterol, 3 were taking fluticasone nasal spray, 4 were taking loratadine, 1 was taking fexofenadine, 1 was taking cetirizine, and 1 was taking benedryl. All participants were at least 45 days past the end of their most recent URI for all of their visits, and were asked to withhold inhaled bronchodilators prior to each study visit: 12 hours for short-acting and 48 hours for long-acting beta agonists. Full analysis was possible in 44 individuals. Exclusion criteria included persistent respiratory symptoms suggesting uncontrolled asthma.

**Table 1:** Baseline demographics and pulmonary functions (mean ± SD) of 45 asthmatic patients

	Non-smoker	Smoker	All
Number of subjects (n)	30	15	45
Male/female	10/20	9/6	19/26
Age (mean ± SD)	38 ± 14	40 ± 13	38 ± 13
Smoking (pack-years)	0	11 ± 10	-
Race (black/white/asian)	20/9/1	10/5/0	30/14/1
PC_20_ (provocative concentration causing a 20 % drop in FEV_1_, in mg/ml)	2.4 ± 4.2	2.6 ± 2.1	2.4 ± 3.6
FEV_1_ (forced expiratory volume in 1-second, in L)	2.7 ± 0.7	2.8 ± 0.8	2.7 ± 0.7
FEV_1_ % predicted (forced expiratory volume in 1-second)	87 ± 10 %	91 ± 13 %	88 ± 11 %
FVC % predicted (forced vital capacity)	90 ± 12 %	97 ± 15 %	92 ± 13 %
FEV_1_/FVC	0.80 ± 0.08	0.78 ± 0.01	0.79 ± 0.08
TLC (total lung capacity, in L)	5.0 ± 1.4	5.1 ± 1.2	5.0 ± 1.4
SVC (slow vital capacity, in L)	3.3 ± 1	3.5 ± 0.9	3.4 ± 1.0
FRC (functional residual capacity, in L)	3.0 ± 0.9	3.3 ± 1.5	3.1 ± 1.1
RV (residual volume, in L)	1.7 ± 0.7	1.7 ± 0.8	1.7 ± 0.7
RV/TLC	0.34 ± 0.09	0.33 ± 0.08	0.34 ± 0.09
Diffusing capacity of carbon monoxide (mL/min/mm Hg)	22.5 ± 5.9	22.9 ± 5.4	22.7 ± 5.6
Specific airway resistance, (in kiloPascals · s)	6.1 ± 3.4	6.0 ± 3.4	6.04 ± 3.3
Exhaled nitric oxide (ppb)	10.4 ± 11	6.15	9.1 ± 10

### Pulmonary function measurements

All subjects underwent baseline spirometry, body-plethysmography, diffusing capacity of the lung for carbon monoxide, and exhaled nitric oxide (eNO) measurements. For spirometry, we used Hankinson's (NHANES III) predicted values [26]. We further made the widely accepted assumption that the reproducibility of FEV_1_ values was 0.1 L [27, 28], so that x-axis changes in Fig. 2a and b between −0.1 and +0.1 L were considered as no effect. In addition, blood was obtained before and immediately after sulforaphane administration to monitor standard blood chemistries for safety (n = 45) and to measures mRNA expression of Nrf2 target genes in peripheral blood mononuclear cells (n = 35).

### Evaluation of beneficial effects of deep inspiration (DI)

DI-induced bronchodilation was performed by using multiple modified single dose MCh challenges on separate days [22, 25, 29]. Briefly, after baseline spirometry, at every single dose challenge, study participants were instructed to abstain from DI for 20 min. At the end of this period, a single dose MCh challenge (starting at 0.025 mg/ml) was delivered with five tidal inspirations from a deVilbiss 646 nebulizer attached to a model 2A Rosenthal-French dosimeter (Laboratory for Applied Immunology, Fairfax, VA). Three minutes later, a single full spirometric maneuver was performed and the degree of airways obstruction was calculated by comparing baseline to post MCh FEV_1_. If the MCh-induced reduction in FEV_1_ was less than 20 %, the participant was asked to return on a separate day for another single dose MCh challenge, using the next highest single dose of MCh (e.g. 0.075 mg/ml). This process was continued with additional single dose challenges (0.25, 0.75, 2.5, 7.5 mg/ml) on separate days, until the single dose inducing ≥20 % reduction in FEV_1_ was achieved. For the challenge at this level, where 20 % or greater reduction in FEV_1_ was obtained, the participant was instructed to continue the procedure by taking 4 DI immediately after the single post-MCh spirometry. Another spirometric maneuver was performed immediately after the 4 DI to calculate the degree to which the participant was able to reverse the MCh-induced airway obstruction (Figure below).
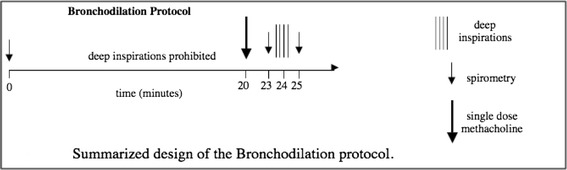


Measuring the difference between the post-MCh FEV_1_ and the FEV_1_ obtained after the 4 DI, we calculated a measure of bronchodilation induced by the DI, which we termed the bronchodilation (BD) index. This measure is calculated as follows:

$$ BD\kern0.5em index=\left(1\mathit{\hbox{-}}\left(\left(1\mathit{\hbox{-}}\left(\left(FE{V}_1\kern0.28em  after\kern0.28em MCh\kern0.28em  and\kern0.28em  after\kern0.28em DI\right)\div \left(FE{V}_1\kern0.5em  baseline\right)\right)\right)\div \left(1\mathit{\hbox{-}}\left(\left(FE{V}_1\kern0.28em  after\kern0.28em MCh\right)\div \left(FE{V}_1\kern0.28em  baseline\right)\right)\right)\right)\right)\times 100 $$

Essentially, the BD index is derived from two components, the reduction in FEV_1_ from baseline after MCh and after DI and the reduction in FEV_1_ from baseline after MCh, but before DI.

On a separate day, the same single MCh dose used to achieve a 20 % or greater reduction in FEV_1_ was again administered after 20 min of quiet breathing, followed by 5 DI to prevent MCh-induced obstruction (Figure below).
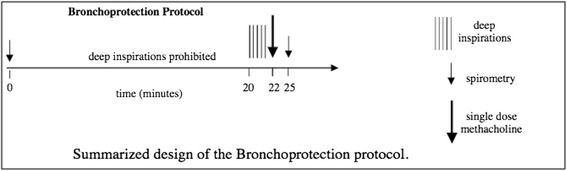


Assessing the difference between the MCh-induced reduction in FEV_1_ on the day no DI were taken to the day on which 5 DI were taken prior to the challenge, we obtained a measure of bronchoprotection induced by the series of DI, which we termed the bronchoprotection (BP) index which has been previously described [22, 29]. This measure is calculated as follows:

$$ BP\kern0.5em index\kern0.5em =\left( 1\mathit{\hbox{-}}\left(\left( 1\mathit{\hbox{-}}\left(\left(FE{V}_1\kern0.5em  after\kern0.5em DIs\kern0.5em  and\kern0.5em  after\kern0.5em MC{h}_B\right)\div \left(FE{V}_1\kern0.5em  baselin{e}_B\right)\right)\right)\div \left( 1\mathit{\hbox{-}}\left(\left(FE{V}_1\kern0.5em  after\kern0.5em MC{h}_A\right)\div \left(FE{V}_1\kern0.5em  baselin{e}_A\right)\right)\right)\right)\right)\times 100 $$

The BP index is derived from two components, the MCh-induced reduction in FEV_1_ from baseline on the day that 5 DI preceded the single-dose MCh challenge and the MCh-induced reduction in FEV_1_ from baseline on the day no DI were taken before the single-dose MCh challenge.

### Acquisition and analysis of CT scans

All scans were performed with a single spiral CT scanner (Siemens, Definition 64) with settings of 120 Kilovolt peak. mAs (milliampere · second) was based on body size (small = 80 mAs, medium = 100 mAs, large = 145 mAs), with a rotation time of 0.5 s, pitch of 1.0 mm, thickness of 0.75 mm, and interval of 0.5 mm. Images were reconstructed using a B35 and B31 algorithm. All subjects were coached and practiced the breathing maneuvers before scanning. For the total lung capacity scans, while in the scanner, all subjects were instructed to take a deep breath and blow it out. This maneuver was repeated three times. On the third deep inspiration, the subjects were instructed to hold their breath. They were then coached to continue to keep holding their breath for the <10 s duration of the scan. For the functional residual capacity (FRC) scans, all subjects were similarly instructed to take a breath in and blow it out. This maneuver was repeated three times. On the third time, the subject was instructed to expel their breath and then hold their breath for the duration of the scan (<10 s). Lung volumes and airway dimensions were calculated using PW software (VIDA Diagnostics, Inc. Coralville, IA) based on the lung CT scans. The PW software calculates the total lung volume, the lung air volume, the lung tissue volume, the lung density in Hounsfield Units (HU), air trapping (% voxels < −856 HU), the luminal diameter, the wall thickness and the wall fraction (wall area/total airway area). In addition, the airways were arbitrarily divided into three groups (small, medium, and large) of a similar number of airways per group.

### Preparation and administration of sulforaphane-rich broccoli sprout extract

The sulforaphane-rich broccoli sprout extract was prepared at the Lewis B. and Dorothy Cullman Chemoprotection Center at Johns Hopkins University, essentially as previously described [30, 31]. We obtained IND approval from the FDA for its use in this study (IND #79274). In brief, specially selected BroccoSprouts™ seeds were surface-disinfected and grown for 3 days in a commercial sprouting facility under controlled light and moisture. A boiling water extract was prepared, filtered, cooled, and treated with the enzyme myrosinase (from daikon sprouts) in order to convert precursor glucosinolates to isothiocyanates, and then lyophilized at a food processing facility (Oregon Freeze Dry, Albany, OR). The lyophilized powder (227 μmol sulforaphane/g powder) was packaged in sterile plastic tubes that were unitized for daily doses by ALFA Specialty Pharmacy (Columbia, MD); each tube contained 100 μmol sulforaphane (440 mg of BSE). The powders (bulk and individual doses) were maintained at −20 °C, repeatedly checked for microbial contaminants and sulforaphane titer before conveyance to the study site to be dispensed to patients.

Study subjects were given both verbal and written instructions to avoid eating any food products that contain the following vegetables (either cooked or raw) until completion of the study: Cruciferous vegetables (such as broccoli, broccolini, broccoli raab, rapini, kale, cabbage, brussels sprouts, cauliflower, arugula, turnips, radish, turnip, turnip greens, kohlrabi, rutabaga, mustard greens, collard greens, chinese cabbage, pak choi, bok choi, napa, watercress, broccoli sprouts, daikon, sauerkraut, coleslaw), vegetables in the onion family (onions, leeks, garlic, or chives), and the following condiments: mustard, horseradish, wasabi, soy sauce, or Worcestershire sauce.

### mRNA gene expression

Nrf2–regulated antioxidative genes were measured in peripheral blood mononuclear cells with quantitative real-time PCR (qRT-PCR). Total RNA was extracted from the cells by use of the Qiagen RNeasy kit (Qiagen, Valencia, CA). Total RNA was used for cDNA synthesis with random hexamers and MultiScribe reverse transcriptase, according to the manufacturer’s recommendations (Applied Biosystems). cDNA (100 ng) was used for quantitative PCR analyses of selected genes: glutamate cysteine ligase catalytic (*GCLC*) and modifier subunits (*GCLM*), NAD()H-quinone oxidoreductase 1 (*NQO1*), and glutathione *S*-transferase-1 (GST1) by using primers and probe sets commercially available from Applied Biosystems. Assays were performed by using the ABI 7000 Taqman system (Applied Biosystems). β-Actin was used for normalization.

**Fig. 1:**
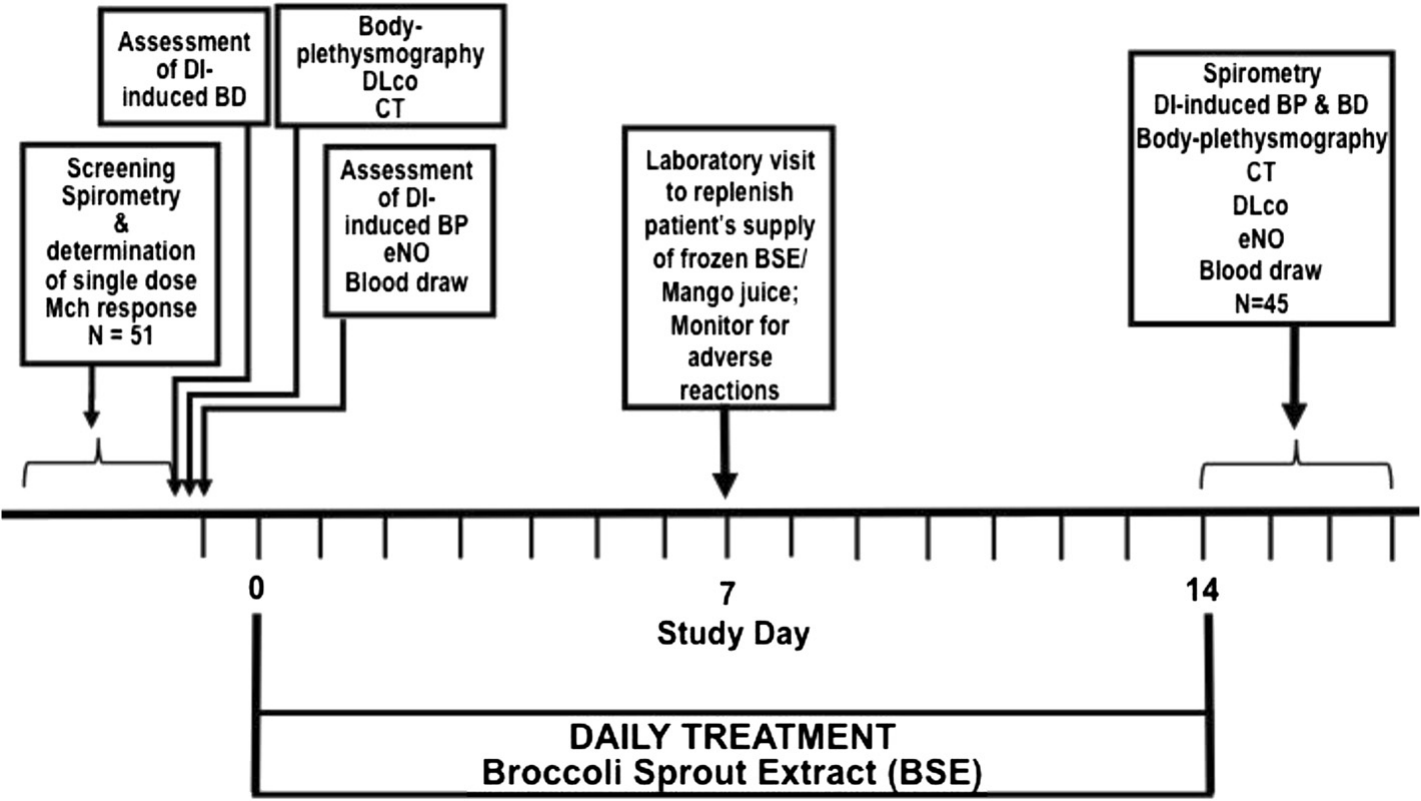
Time line of trial protocol

After the baseline assessment of BP and BD, spirometry, body-plethysmography, diffusion capacity of carbon monoxide, exhaled NO, and CT scan measurements were completed (Fig. 1), volunteers received a 1-week supply of 20-ml bottles containing 100 μmol of sulforaphane each dissolved in mango juice, which masks the pungency of the BSE [20], to be stored in a freezer. Each morning, a single bottle was allowed to thaw at room temperature until consumption of the contents in the evening. The subject then received a second 7-day supply from the laboratory, and the procedure was repeated. At the end of this period, BP, BD, CT scan, spirometry, body plethysmography, diffusion capacity of carbon monoxide, and exhaled NO were re-assessed within 3 days of consumption of the last bottle (Fig. 1). In total, the subjects consumed 100 μmol of sulforaphane per day on 14 consecutive evenings.

### Safety and adverse event monitoring

Adverse event monitoring and documentation by severity, duration, and relatedness were performed by the study physician at each visit. Standard laboratory chemistries were drawn at baseline and after the two weeks of consuming sulforaphane. Study stopping rules were based on the standard practice of our local IRB and the FDA. Study drug safety and adherence to the protocol were monitored annually by the IRB.

### Statistics

Data analysis was performed with JMP 11.0.0 software (SAS Institute, Cary, NC). To compare the demographic and baseline pulmonary function data, one-way ANOVA and Chi-squared tests were used. To compare the effects of sulforaphane on changes in BP and BD, lung function, CT measurements, oxidative stress genes, and effects of smoking, matched-pairs comparisons (pre- and post-sulforaphane) with non-parametric Wilcoxon Sign Rank Analyses were performed. In addition, one-way ANOVA, and simple linear regressions were used where indicated. To examine the effects of specific genes and changes in FEV_1_ interaction on the BP, we constructed a multivariate regression model. Significance was assumed at p ≤ 0.05.

## Results

The time-line of the protocol for this study is presented in Fig. 1.

### Screening and selection of asthmatic subjects

A total of 51 individuals with airway hyperresponsiveness were screened. The demographic and baseline pulmonary functions as shown in Table 1. Full analysis was possible in 44 individuals.

### Primary outcomes

The primary goal of these studies was to determine whether sulforaphane administration to 44 asthmatic patients: (i) affected the magnitude of the bronchoconstrictor effect of single doses of MCh challenge, and (ii) affected the protective effects of deep inspiration on the magnitude of the bronchoconstrictor effect of MCh challenge. The final metric in all these studies was FEV_1._

**Fig. 2:**
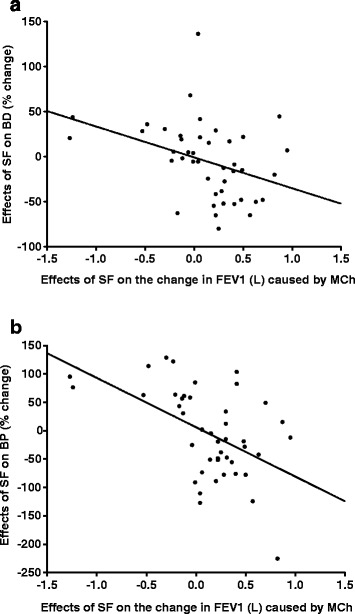
Negative correlation between the changes (%) resulting from sulforaphane (SF) administration on: **a**. bronchodilation (BD) and on **b**. bronchoprotection (BP) in asthmatic subjects, and the effects of sulforaphane administration on the reduction of FEV_1_ caused by MCh challenge. **a**: There was a significant negative correlation between the changes in BD and the changes in FEV_1_ (r^2^ = 0.13, p = 0.01). As the decrease in FEV_1_ with MCh challenge (airway narrowing) became larger with administration of sulforaphane, the BD response became smaller. **b**: There was a significant negative correlation between the changes in BP and the changes in FEV_1_ (r^2^ = 0.26, p = 0.0005). As the decrease in FEV_1_ with MCh challenge (airway narrowing) became larger with administration of sulforaphane, the BP response became smaller. The Mean control FEV_1_ was 2.7 ± 0.7 L, and a single MCh challenge caused a reduction in FEV_1_ by 0.78 ± 0.3 L. We further made the widely accepted assumption that the reproducibility of FEV_1_ values was 0.1 L, so that x-axis changes in Fig. 2a and b between −0.1 and +0.1 L were considered as no effect

The mean reduction in FEV_1_ resulting from a single dose MCh challenge was 28.7 ± 7.2 % (mean ± SD) (Table 2), but individual asthmatics varied markedly in their responses to sulforaphane (Fig. 2a and b). Whereas in 60 % of the asthmatics sulforaphane blocked the bronchoconstrictor effects of MCh challenge, in 20 % of these subjects sulforaphane aggravated the bronchoconstrictor effect of MCh, and in a similar proportion (20 %) it had no effect.

**Table 2:** Effects of sulforaphane (SF) administration and deep inspiration on **A.** Changes in forced expiratory volume in 1-second (FEV_1_) produced by the bronchoconstrictor MCh, and **B.** Magnitudes of bronchoprotection (BP) and bronchodilation (BD) effects

A. Treatment	% Change in FEV_1_ (mean ± SD)^a^		
	Before SF	After SF	p for SF effect
MCh	−28.7 ± 7.2	−22.7 ± 12.7	0.006
MCh preceded by 4 deep inspirations (*for BP*)	−23.0 ± 13.4	−19.1 ± 11.9	0.04
MCh followed by 4 deep inspirations (*for BD*)	−18.4 ± 7.7	−16.3 ± 10.7	0.34
B. Magnitudes of deep inspiration effects	Change in BP and BD (mean ± SD)		
	Before SF	After SF	p for SF effect
BP	15.5 ± 52.7	10.3 ± 57.1	0.83
BD	32.5 ± 32.7	26.7 ± 30.6^a^	0.33

^a^One value was outside the acceptable range (>2 S.D.) and has been censored

When the change in FEV_1_ response to MCh was analyzed post hoc as either a decrease or an increase in response to sulforaphane administration, the resulting BP and BD responses were dramatically different. Those subjects whose FEV_1_ response to MCh decreased (−13 ± 8 %), had a concomitant reduction in BP response (p = 0.002; Table 3), whereas those subjects whose FEV_1_ response to MCh increased (11 ± 11 %), experienced an improvement in BP (p = 0.004, Table 3). Changes in BD paralleled these changes in BP, (p = 0.02 for the subjects whose FEV_1_ response to MCh decreased and p = 0.04 for the subjects whose FEV_1_ response to MCh increase; Table 3). The implication of these paradoxical responses is not yet clear and the mechanism is not understood.

**Table 3:** The BP and BD responses to sulforaphane treatment, classified according to initial increased or decreased forced expiratory volume in 1-second (FEV_1_) response to MCh (mean ± SD). Wilcoxon sign rank test

	BP			BD		
	Before SF	After SF	p-for SF effect	Before SF	After SF	p-for SF effect
Overall FEV_1_ (from Table 2)	15.5 ± 52.7	10.3 ± 57	0.82	32.5 ± 32.7	28.2 ± 28	0.33
Decreased FEV_1_ response to MCh (n = 29)	37.6 ± 38.0	−0.8 ± 64	0.002	38.3 ± 34.6	23.2 ± 35.1	0.02
Increased FEV_1_ response to MCh (n = 15)	−27.2 ± 51.7	31.7 ± 33	0.004	19.6 ± 25.1	33.4 ± 18.6	0.04

### Secondary outcomes

Secondary outcomes examined changes in pulmonary function (Table 4) and lung morphology as determined by high-resolution CT scans (Table 5). We found a significant reduction in specific airway resistance (p = 0.03, Table 4), and a small but significant increase in the small and medium airway luminal area (p = 0.03, Table 5).

**Table 4:** Change in pulmonary function with sulforaphane (SF) treatment (mean ± SD)

	Before SF	After SF	p for SF effect
FEV_1_ % predicted (forced expiratory volume in 1-second)	88 ± 11 %	88 ± 11 %	0.79
FVC % predicted (forced vital capacity)	92 ± 13 %	92 ± 11 %	0.95
FEV_1_/FVC	0.79 ± 0.08	0.77 ± 0.01	0.56
TLC (total lung capacity, in L)	5.0 ± 1.4	4.9 ± 13	0.10
SVC (slow vital capacity, in L)	3.4 ± 1.0	3.3 ± 0.9	0.38
FRC (functional residual capacity, in L)	3.1 ± 1.1	3.1 ± 1.0	0.56
RV (residual volume, in L)	1.7 ± 0.7	1.6 ± 0.7	0.56
RV/TLC	0.34 ± 0.09	0.34 ± 0.1	0.97
Diffusing capacity of carbon monoxide, (mL/min/mm Hg)	22.7 ± 5.6	22.4 ± 5.7	0.46
Specific airway resistance (kiloPascals · s)	6.04 ± 3.3	4.8 ± 2.5	0.008
Exhaled nitric oxide (ppb)	8.8 ± 10	9.3 ± 10.9	0.80

**Table 5:** CT scan measurements of changes in volume, luminal area and airway wall thickness with sulforaphane (SF) treatment measured at either total lung capacity (TLC) or functional residual capacity (FRC) (mean ± SD)

	Before SF	After SF	p for SF effect
TLC lung volume (mL)	4175	4195	0.68
FRC lung volume (mL)	1470	1647	0.44
Mean lung density at TLC (HU)	−820 ± 34	−823 ± 33	0.13
Air trapping (%) (FRC)	4.6 ± 8.3	7.9 ± 11.8	0.10
Airway luminal area (TLC) mm^2^	44.7 ± 10.3	45.1 ± 8.5	0.07
Large	103 ± 13	102.9 ± 16	0.73
Medium	30.9 ± 1.8	32 ± 4.5	0.04
Small	13.1 ± 1.6	14.3 ± 3.4	0.01
Airway wall thickness (fraction luminal diameter) (TLC)	0.57 ± 0.03	0.57 ± 0.02	0.82
Large	0.45 ± 0.02	0.46 ± 0.03	0.12
Medium	0.58 ± 0.02	0.58 ± 0.03	0.96
Small	0.67 ± 0.02	0.66 ± 0.04	0.03

### Gene-dose effects

Peripheral blood mononuclear cells were obtained from 35 subjects for the measurement of Nrf2–regulated antioxidant genes, and we were able to compare 28 sample pairs before and after sulforaphane treatment. When gene activities were classified according to the changes in FEV_1_ (either a decrease or an increase response to MCh after sulforaphane administration), the results were very different. When the FEV_1_ response to MCh decreased after sulforaphane administration, the activities of all three Nrf2-regulated genes (NQO1, HO1, GCLM) decreased. In contrast, when the FEV_1_ response to MCh increased after sulforaphane administration, the gene activities of all three genes increased (Table 6). Comparing the changes in each gene expression with changes in FEV_1_ response to sulforaphane treatment, the differences in gene expression was significant for GCLM (p = 0.03) and NQO1 (p = 0.047).

**Table 6:** Change in oxidative stress gene expression related to changes in Forced Expiratory Volume in 1 second (FEV_1_) in response to single dose MCh challenge (mean ± SD). P-value for pre vs. post sulforaphane

	Gene Expression (% change from baseline)		
	GST1	NQO1	GCLM
Decreased FEV_1_ response to MCh	−6.11 ± 27.0	−73.1 ± 255.4	−19.2 ± 40.6
	p = 0.67	p = 0.23	p = 0.03
Increased FEV_1_ response to MCh	11.8 ± 56.0	141.9 ± 283.7	32.4 ± 75.0
	p = 0.38	p = 0.047	p = 0.58

### Bronchoprotection (BP), bronchodilation (BD), and gene expression

The BP and BD responses were also analyzed according to changes in gene expression (either a decrease or an increase resulting from sulforaphane administration). There was a significant relationship between the change in NQO1 gene expression and BP (p = 0.03). When NQO1 decreased after sulforaphane administration, the BP worsened (−54.4 ± 78.3 %) and when NQO1 increased after sulforaphane administration, the BP improved (20.6 ± 72.8 %). There were no statistically significant relationships for the other genes or for BD.

We next examined the potential interactions among these factors. A multivariate regression model was constructed using the change in BP as the outcome variable. Independent variables were the changes in MCh-induced decrease in FEV_1_ (the difference between baseline before and after sulforaphane treatment), and the change in GCLM, GST1, and NQO1 gene expression. The overall model was significant (r^2^ = 0.67, p = 0.0003). Controlling for the other variables, there was a significant negative correlation between the change in BP and the change in FEV_1_ from baseline (p = 0.002), and a significant positive correlation between the change in BP and the change in NQO1 gene expression (p = 0.007).

### Bronchoprotection, bronchodilation, and smoking history

When BD and BP responses in the non-smokers and smokers were partitioned by the change in FEV_1_ response to MCh, they mirrored the overall changes. In those subjects whose FEV_1_ response to MCh decreased whether they were non-smokers or smokers, there was a concomitant reduction in BP and BD responses (Table 7). Conversely, subjects whose FEV_1_ response to MCh increased whether they were non-smokers or smokers, showed improvement in BP and BD values (Table 7).

**Table 7:** BP and BD responses to sulforaphane (SF) arranged according to changes in forced expiratory volume in 1-second (FEV_1_) to MCh (mean ± SD). A. Non-smokers (n = 30), B. Smokers (n = 15).

A. Non-smokers						
	BP (% change)			BD (% change)		
	Before SF	After SF	p for SF effect	Before SF	After SF	p for SF effect
Decreased FEV_1_ response to MCh	40.4 ± 40.4	−3.8 ± 46.8	0.002	33.7 ± 39.4	23.8 ± 36.8	0.20
Increased FEV_1_ response to MCh	−49.3 ± 29.7	23.5 ± 35.8	0.004	17.7 ± 27.0	36.3 ± 17.7	0.04
B. Smokers						
	BP (% change)			BD (% change)		
	Before SF	After SF	p for SF effect	Before SF	After SF	p for SF effect
Decreased FEV_1_ response to MCh	32.3 ± 34.2	5.1 ± 91.1	0.28	47.1 ± 21.8	22.1 ± 33.4	0.06
Increased FEV_1_ response to MCh	17.1 ± 60.7	48.1 ± 20.6	0.44	22.3 ± 23.1	27.4 ± 20.1	0.63

### Safety and tolerance

Sulforaphane treatment was safe and well-tolerated. Inspection of the laboratory results among all the subjects showed that only one set of laboratory values in one subject showed a significant liver function (transaminase) elevation after sulforaphane administration. On further questioning, the subject reported that he had run a marathon the day before the blood draw after sulforaphane treatment, and this may have been responsible for changes in liver enzymes. On repeating the blood chemistries, one week later, all blood chemistries were within normal limits.

## Discussion

To our knowledge, this is the first study that has examined the effects of the phytochemical sulforaphane on the response to DI in individuals with asthma. This study addressed the hypothesis that oxidative stress and inflammation play roles in the mechanisms of the beneficial effects of DI. While our a priori primary outcome was negative, post hoc analysis examining the groups according to whether they had an increase or a decrease in FEV_1_ response to Mch told a very different story.

When sulforaphane attenuated the decline in FEV_1_ following a single MCh challenge, BP worsened. In contrast, if the FEV_1_ response to a single MCh challenge remained the same or increased slightly after sulforaphane treatment, BP improved dramatically. There were similar changes for the BD responses. In addition when sulforaphane increased NQO1 gene expression, there was an associated increase in FEV_1_ response to MCh and independently associated improvement in the BP response of DI in this cohort of asthmatics.

It is predictable that there should be a range of sulforaphane responses among individuals, and that they be related to some other biological phenomenon that we have yet to understand. For example, we have observed striking demonstrations of this effect in our clinical studies of sulforaphane effects on autism spectrum disorder behavioral metrics in young men [31], and in clinical evaluation of the effect of sulforaphane on both aflatoxin and air pollutant metabolites [30, 32].

It is not yet fully explainable (although expected), that multiple mechanisms could be at play in creating this range of responses. The literature suggesting a bi-phasic response, commonly referred to as the U-shaped or J-shaped curve, or hormesis [33, 34] and well described in the context of phytochemicals [35], points to many such examples in which a compound may have a protective or therapeutic effect at one concentration and a toxic or detrimental effect at another higher level of exposure. This is well demonstrated in the case of Nrf2-active compounds, in particular in their effects on redox signaling, and differential pro- and anti-oxidant activities that are concentration dependent [36].

A limited number of animal and human studies have examined the effects of sulforaphane on airway responsiveness. Park and colleagues showed that sulforaphane inhibits ovalbumin-induced airway inflammation in a murine model of asthma [37]. They further surmised that this occurred though suppression of GATA-3 and SOCS-3 and increased T-bet and SOCS-5 expression. Whether this also occurs in humans has not been determined. Nrf2–antioxidant response element binding was reduced in cultured airway smooth muscle cells from patients with severe asthma compared to similar cells from patients with moderate asthma or with those from normal subjects [38]. How this affects the asthmatic response in vivo is unknown.

Sulforaphane effects on upper airway inflammation, and on airway response to challenge, have also been measured. Oral sulforaphane induced airway Phase 2 antioxidant enzyme expression in a dose-dependent fashion in exfoliated human upper airway cells [39]. In a follow-up study, when sulforaphane in the form of a broccoli sprout extract was administered orally for 4 days before a diesel exhaust particle challenge, total cell count in the nasal lavage was reduced by more than 50 % [40]. These studies establish that the oral administration of sulforaphane is an effective means of inducing cytoprotective enzymes in the upper airway and by inference, probably also in the lower airway. In addition, the doses in these published studies were similar to those used in the current study and thus support our choice of dosing regimen. We chose to administer the sulforaphane for a longer period of time, 14 days rather than 4 days, in order to be confident that enough time had elapsed for substantial enzyme induction.

It is well recognized that pro-inflammatory cytokines, chemokines, as well as other cell mediators, play important roles in the allergic inflammatory process in asthma. Dysfunction of the Nrf2 pathway has also been linked to severe allergen-driven airway inflammation and hyper-responsiveness in mice [6]. Disruption of the Nrf2 signaling pathway in mouse models has also been directly linked to chronic inflammation such as that associated with asthma [6]. As a potent inducer of cytoprotective enzymes (antioxidant and anti-inflammatory) via Nrf2, sulforaphane thus directly affects both the antioxidant deficiencies that have been associated with poor asthma control and accelerated lung function decline [16–18], and the inflammatory component of these conditions [12–14], both of which represent attractive therapeutic targets.

Upregulation of Phase 2 enzymes and of other Nrf2 targets by sulforaphane has previously been shown in vitro and in animal models [9, 39, 41–43]. The use of broccoli sprout extract as a vehicle for well-quantified oral delivery of sulforaphane permitted us to examine the role of oxidative stress mediated through the Nrf2-signaling pathways on lung function in asthma. Edible plants contain a wide variety of phytochemicals, some of which are phase 2 enzyme inducers. Large quantities of inducers of enzymes that are potent antioxidants can be delivered in the diet by small quantities of broccoli sprouts (e.g., three-day-old sprouts) that contain as much inducer activity as 10–100 times larger quantities of mature broccoli. A well-characterized, stable, clean, standardized, homogeneous, sulforaphane-rich food extract can be produced and utilized across a range of cell culture, animal, and clinical studies, permitting direct comparison of results. Thus, a large body of evidence has accumulated around the world in a variety of experimental systems suggesting that sulforaphane is the active agent in broccoli sprout extracts responsible for almost all of the phase 2 response that is induced following treatment with broccoli sprout extracts. Quantifiable and reproducible extraction of sulforaphane from BSE has thus permitted us to target specifically the Nrf2 pathway in order to elucidate the mechanisms of oxidative stress in patients with asthma.

Few studies have examined the consumption of foods rich in antioxidants in general and sulforaphane specifically, on airway reactivity and asthma. Early studies suggested that changes in the diets of children was a determinant of the worldwide increases in asthma and allergies [44], and that infrequent consumption of fresh fruit was associated with impaired lung function [45]. More recently, intake of some vegetables was also associated with reduced prevalence of adult asthma [46]. Commonly consumed fruits, vegetables, and nuts, and high adherence to a traditional Mediterranean diet during childhood were shown to have beneficial effects on symptoms of asthma and rhinitis [47]. Adherence to a Mediterranean diet during pregnancy also had a protective effect against asthma-like symptoms and atopy in childhood [48]. An intervention that administered a food plant, (*Moringa oleifera*) known to be rich in glucosinolates similar to the precursor of sulforaphane, to 20 asthmatic subjects daily for 3 weeks, significantly improved symptom severity, which was accompanied by improvements in FEV_1_, FVC, and peak expiratory flow rate [49]. Most impressively, a new meta analysis of 12 cohort, 4 population-based case–control, and 26 cross-sectional studies came to the conclusion that reduced risk of asthma in adults and children was associated with higher intake of fruit and vegetables (RR = 0.54; 95 % CI, 0.41-0.69) [50].

The relationship between DI, airway mechanics, and airway hyperresponsiveness has been a focus of asthma research for over 2 decades [24]. Several earlier studies have shown that DI has beneficial effects on human airways [51, 52]. In healthy individuals, DI taken before exposure to MCh protected the airways from bronchoconstriction [53]. This property is largely lost in asthma, even in mild disease (5). Deep inspirations can also reverse airway obstruction that has been experimentally induced with a direct spasmogen [54]. The bronchodilatory effect of DI is minimally affected in mild asthmatics (5), but decreases with increasing severity of asthma [55], raising the possibility that the impairment of this physiologic function of the lung is one of the determinants of severe obstructive disease.

Our findings are consistent with previous demonstrations that BP and BD effects of DI become more prominent when the MCh-induced bronchoconstriction in the absence of DI is of substantial magnitude [25]. Our findings are also consistent with the dependence of the effects of DI on the magnitude of bronchoconstriction (Fig. 2). When the responses of our subjects were evaluated by group: (subjects with either a decrease or an increase in response to MCh), the BD and BP outcomes were dramatically different (Table 3). To our knowledge, this is the first demonstration of a decrease in response to MCh leading to a worsening of the BD and BP responses and not simply a diminution of the response amplitude [25]. Why this occurred and whether the effect of sulforaphane on the oxidative stress balance was involved will require further exploration.

Among nonsmokers, both BP and BD decreased in those subjects with a decreased FEV_1_ response to MCh, (Table 7). In contrast, if they had no change, or any increase in their FEV_1_ responses to MCh, both BD and BP improved significantly. There were similar trends for smokers, but the changes in BD for subjects with a reduced FEV_1_ response to MCh was significantly worse than those who had non- or marginal FEV_1_ responses to MCh. Since changes were of similar magnitude in both smokers and nonsmokers, the difference in the significance of that effect may reflect the lower number of smokers enrolled in the study. Based on animal studies, a greater effect of sulforaphane on BD and BP would be expected in smokers. Stimulation of Nrf2 pathways has been shown to protect against the harmful effects of cigarette smoke (which releases large quantities of free radicals and increases lung levels of reactive oxygen and nitrogen species (ROS and RNS) in murine models [56].

Among all the subjects, there was a significant decrease in specific airway resistance (Table 4) with the administration of sulforaphane. This is likely explained by the concomitant increase in small and medium airway luminal area (Table 5) after sulforaphane administration. While these changes did not translate into an improvement in the more commonly used lung function measurements such as FEV_1_, FVC, and FEV_1_/FVC, it does suggests an overall beneficial effect of sulforaphane on the airways of asthmatic individuals.

In conclusion, administration of sulforaphane, a potent upregulator of genes protecting against oxidative stress and inflammation, may be useful both to determine the mechanisms that lead to asthma, as well as suggesting a potential therapy to treat asthma. Sulforaphane administration improved the BP response in asthmatics who had an increase in NQO1 gene expression and did not have a decrease in their initial response to the MCh challenge. Therefore, sulforaphane administration was able to improve a major defect of even mild asthma. This should encourage further examination of this major cytoprotective signaling pathway as a potential mechanistic approach to the treatment or prevention of asthma. Furthermore, since the administration of anti-inflammatory drugs do not completely prevent the symptoms or progression of the disease in a substantial subset of asthmatics, these findings also raise the possibility of the use of sulforaphane, or foods rich in sulforaphane, as potential adjuvant treatments for asthma.

## Abbreviations

BD: Bronchodilation
BP: Bronchoprotection
BSE: Broccoli sprout extract
DI: Deep inspiration
ERV: Expiratory reserve volume
FEV_1_: Forced expiratory volume in 1 second
FRC: Functional residual capacity
GST: Glutathione transferase
GCLM: Glutamine-cysteine ligase, regulatory subunit
IC: Inspiratory capacity
MAPK: Mitogen-activated protein kinase
MCh: Methacholine
NfκB: Nuclear factor kappa-light-chain-enhancer of activated B cells
NQO1: NAD(P)H, Nicotinamide-quinone oxidoreductase 1
Nrf2: Nuclear factor erythroid-related factor 2
PBMC: Peripheral blood mononuclear cells
PC_20_: Provocative concentration causing a 20 % drop in FEV_1_
RV: Residual volume
sRAW: specific airway resistance
SVC: Slow vital capacity
TGV: Thoracic gas volume
TLC: Total lung capacity
TVC: Total vital capacity
